# Electrochemical Enzyme Biosensor Bearing Biochar Nanoparticle as Signal Enhancer for Bisphenol A Detection in Water

**DOI:** 10.3390/s19071619

**Published:** 2019-04-04

**Authors:** Yang Liu, Lan Yao, Lingzhi He, Na Liu, Yunxian Piao

**Affiliations:** Key Laboratory of Groundwater Resources and Environment (Jilin University), Ministry of Education, Jilin Provincial Key Laboratory of Water Resources and Environment, College of New Energy and Environment, Jilin University, Changchun 130021, China; yangliu16@mails.jlu.edu.cn (Y.L.); yaolan17@mails.jlu.edu.cn (L.Y.); helz17@mails.jlu.edu.cn (L.H.); liuna@jlu.edu.cn (N.L.)

**Keywords:** biochar nanoparticle, enzyme, bisphenol A, biosensor

## Abstract

An electrochemical tyrosinase enzyme (Tyr) biosensor using a highly conductive sugarcane derived biochar nanoparticle (BCNP) as a transducer and signal enhancer (BCNPs/Tyr/Nafion/GCE) was developed for the sensitive detection of bisphenol A (BPA). The BCNPs/Tyr/Nafion/GCE biosensor exhibited improved amperometric current responses such as higher sensing signal, decreased impedance and lowered reduction potential compared with the Tyr/Nafion/GCE due to high conductivity property of the biochar nanoparticle. Under the optimized conditions, it could detect BPA in good sensitivity with linear range from 0.02 to 10 μM, and a lowest detection limit of 3.18 nM. Moreover, it showed a low *K*_m_ value, high reproducibility and good selectivity over other reagents, and the BCNPs/Tyr complex solution also showed good stability with 86.9% of sensing signal maintained after one month storage. The biosensor was also successfully utilized for real water detection with high accuracy as validated by high performance liquid chromatography. Therefore, the biochar nanoparticle based enzyme biosensor proved to be a potential and reliable method for high performance detection of pollutants in the environment.

## 1. Introduction

Bisphenol A (2, 2-bis(4-hydroxyphenyl)propane, BPA) is a phenolic compound with high production volume widely used in the plastic industry, in particular as a monomer for producing polycarbonate plastics (PC) and epoxy resins [1,2,3]. It is also an endocrine disrupting compound, has estrogenic activity, and may have adverse effects on humans, wildlife, and reproductive systems [4]. BPA has been detected in many areas, such as freshwater, seawater, landfill, sludges, air, dust particles [5,6], and even in food matrices and drinking water [7,8]. The predicted no-effect concentration of BPA on the scale of around several micrograms per liter [9,10]. Therefore, it is necessary to develop a sensitive, reliable and simple method to detect BPA.

A variety of analysis methods for BPA detection have been used, such as liquid chromatography–mass spectrometry [11], liquid chromatography–tandem mass spectrometry [12], gas chromatography–mass spectrometry [11,13], liquid chromatography–coulometric detection [14], liquid chromatography–fluorescence detection [15,16] and liquid chromatography–UV detection [17]. These methods have high sensitivity and reliability, however, most of them are time consuming, cannot be performed on-site and require professional and technical personnel. To overcome these problems, a series of new methods have emerged including the fluorometric method [18,19], enzyme linked immunosorbent assays [20] and so on. Among these, the electrochemical methods [21], electrochemical enzyme biosensors in particular have drawn great attention due to their simplicity, sensitivity, selectivity, low cost, and field measurement capabilities.

It is well known that sensing materials play an extremely important role in the preparation of enzyme biosensors. They are the core of enzyme biosensors because they not only improve the catalytic activity and stability of the enzyme, but also enhance the sensitivity of the biosensors. As a new material, biochar has gained very much attention in this capacity. Biochar is the carbon-rich material usually obtained through the heating of biomass, such as wood, manure or leaves in a closed container with little or no available air [22]. There are some essential advantages of biochar, such as high surface area due to the unique porous structure, high cost effectiveness, sustainability, and an easy production process which allows the production of materials with extensive applications at a lower cost compared to materials from petrochemical or other chemical processes [23]. Furthermore, the source of biochar is ubiquitous, and plant derived biochar may provide a biocompatible environment for the stable accommodation of biomaterials such as microorganisms and enzymes [24]. For years, biochar was mainly used as a contaminant adsorbent, and also used in soil amendment to mitigate greenhouse gas emissions and improve soil quality. Recently, it was also utilized as a precursor for making catalysts, and electrode materials in microbial fuel cells and sensors [23,25,26,27,28,29,30,31,32,33]. However, the utilization of highly conductive biochar derived nanoparticles for the construction of electrochemical enzyme biosensors has not yet been extensively studied. 

Recently, we demonstrated some attractive characteristics of biochar that are beneficial for biochar based electrochemical sensor development. These include that nanometer-sized biochar particles denoted as biochar nanoparticles (BCNPs) have the greater surface area vs. the conventional millimeter sized biochar particles [34] and that biochars prepared at high pyrolysis temperature have a great electrochemical catalytic property [35,36,37]. In this sense, an enzyme biosensor prepared by using such biochar nanoparticles with outstanding electrochemical catalytic properties and high surface area would have the potential to improve detection sensitivity. 

In this study, a tyrosinase enzyme biosensor with conductive biochar nanoparticles was constructed for bisphenol A determination in water. Tyrosinase as an ortho-hydroxylation oxidase was used as the signaling enzyme because it possesses catalytic bioactivity for BPA that can be oxidized sequentially to polyhydric phenol and the electrochemically active o-quinone [38,39,40], where the o-quinone further can be reduced at the electrode surface producing reducing current signal proportional to concentrations of BPA. The sensitivity, kinetic parameters, reproducibility and selectivity of the novel BCNP enzyme biosensor were explored. The applicability of the biosensor for real environmental detection was also well demonstrated using groundwater. Because of the great electrochemical properties of BCNP and the high catalytic activity of the loaded enzyme, sensitive and rapid detection of BPA pollutant would be enabled.

## 2. Materials and Methods

### 2.1. Chemicals and Apparatus

Bisphenol A (BPA), K_3_Fe(CN)_6_, a Nafion solution (~5% in a mixture of lower aliphatic alcohols and water) and absolute ethanol were purchased from Sigma Aldrich (St. Louis, MO, USA). Tyrosinase (from mushroom, ≥1000 unit mg^−1^, PI 5.92) were also purchased from Sigma. Multi-wall carbon nanotubes abbreviated as MWNTs (diameter <8 nm; purity >95%; length, 10–30 μm) were obtained from the XFNANO. Graphite Nanopowder abbreviated as GN (average particle size <100 nm; purity >95%) were obtained from the SkySpring Nanomaterials, Inc. Graphene abbreviated as GP were obtained from the Shenzhen Turing Evolution Technology Co., Ltd, Shenzhen, China. All other reagents were analytical grade. A 50 mM phosphate buffer solution (pH 7.0, PB) consisting of Na_2_HPO_4_·12H_2_O and NaH_2_PO_4_·2H_2_O was used as electrolyte throughout the experiments unless addressed. 

The scanning electron microscope images were obtained using a XL-30 field emission scanning electron microscope (FEI, Hillsboro, TX, USA). Fourier Transform Infrared (FTIR) spectra were obtained by using a Spectrum GX apparatus (Nicolet Company, Madison, WI, USA) with the medium infrared range (4000–1000 cm^−1^) and a spectral resolution of 4 cm^−1^. For FTIR analysis, the Tyr, BCNPs and BCNPs/Tyr in double distilled water were dried at 30 °C for about 3 h, after which the dried samples were mixed with KBr powder, followed by the production of pellets with a tableting machine (FW-4, Tianguang Optical Instrument Co., Ltd., Tianjin, China). Circular dichroism (CD) spectroscopy was performed by a CD Spectropolarimeter (Jasco-810, Tokyo, Japan) at room temperature using a quartz cuvette with 1.0 mm path length in a wavelength range of 190–260 nm. The specific surface areas of the materials were measured by the Brunauer–Emmett–Teller (BET) method (ASAP 2020).

### 2.2. Preparation of Biochar Nanoparticles

High conductivity biochar nanoparticles were prepared as previously reported [24]. Briefly, the dried bagasse was pyrolyzed at a temperature of 800 °C under the anoxic condition in a high temperature furnace (OTF-1200X, Kejing Material Technology Co., Ltd., Hefei, China). The obtained biochar was groud in an agate mortar to be finely adherent and dissolved in ultrapure water. After ultrasound sonication and centrifugation (5000 rpm, 3 min) processes, the centrifuged supernatant was filtered in a suction filtration device (filter pore size of 220 nm), and the obtained biochar nanoparticles on the filter were dried for further use. 

### 2.3. Preparation of BCNP Enzyme Biosensor

**Scheme 1:**
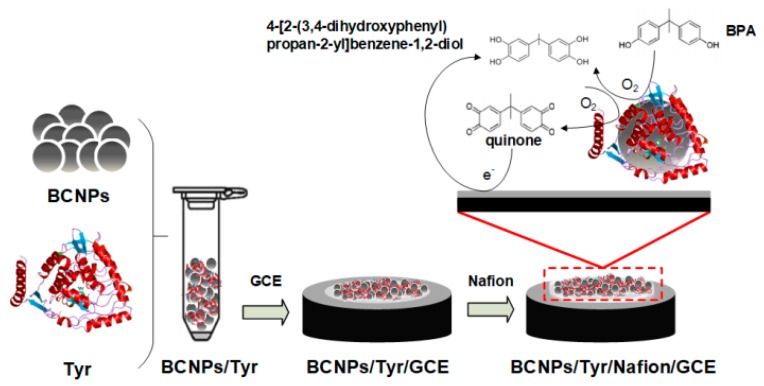
Schematics of the procedures for the preparation of the BCNPs/Tyr/Nafion/GCE biosensor and the principle of electrochemical BPA sensing.

A glassy carbon electrode (GCE) with a 3 mm diameter (Shanghai Chenhua Instrument Co. Ltd., Shanghai, China) was polished with 0.5 µm alumina slurry, and cleaned with absorbent cotton to remove excess alumina slurry. After sonication for 4 min, it was washed with ultrapure water and methanol, and dried under a nitrogen gas. For the preparation of the BCNP enzyme biosensor, 0.5 mg mL^−1^ of tyrosinase was mixed with 0.375 mg mL^−1^ of BCNPs in the phosphate buffer solution (50 mM, pH 7.0). Then, the composite mixture (5.4 μL) of BCNPs and enzyme was cast onto the pristine GCE electrode, followed by adding 0.6 μL of Nafion (0.5 wt%) (Scheme 1). Then, it was dried under an ambient condition and denoted as BCNPs/Tyr/Nafion/GCE. 

Other electrodes such as GN/Tyr/Nafion/GCE, MWNTs/Tyr/Nafion/GCE and GP/Tyr/Nafion/GCE were also prepared with GN, MWNTs and GP using the same procedures for preparation of the BCNPs/Tyr/Nafion/GCE. 

### 2.4. Electrochemical Measurements

Electrochemical measurements were performed using a CHI660E electrochemical workstation (Shanghai Chenhua Instrument Co. Ltd., Shanghai, China). A three-electrode system was used for electrochemical detection where the BCNPs/Tyr/Nafion/GCE electrode, Ag/AgCl electrode, and platinum wire electrode were used as working, reference, and auxiliary electrodes, respectively. Electrochemical BPA detection was performed in a 10 mL PB solution. The cyclic voltammetry was performed at the potential range of −0.3 to 0.4 V with scan rate of 50 mV s^−1^. The electrochemical impedance spectroscopy (EIS) measurements were carried out in a 5 mM K_3_Fe(CN)_6_ solution containing 0.1 M KCl and the frequency range was from 0.01 to 100,000 Hz. 

### 2.5. Real Environmental Water Sample Detection

A ground water sample was obtained from the surrounding area of Jiefang Road, Chaoyang District, Changchun, China. Without any further treatment, the ground water sample was diluted 100-fold with PB solution and spiked with BPA to specific concentrations. The amounts of BPA were amperometrically analyzed by using the BCNPs/Tyr/Nafion/GCE biosensor.

The BPA concentration was also detected using the HPLC method with an LC-20AT HPLC system (Shimadzu, Kyoto, Japan) that was installed with a UV-vis detector at 278 nm and an Eclipse Plus C18 column (4.6 mm × 150 mm, particle size of 5 µm, Agilent, Santa Clara, CA, USA). The mobile phase consisted of acetonitrile and ultrapure water at a volume ratio of 1:1, and the flow rate was set to 1.0 mL min^−1^ [41].

## 3. Results and Discussion

### 3.1. Characterization of BCNP Enzyme Biosensor

**Figure 1:**
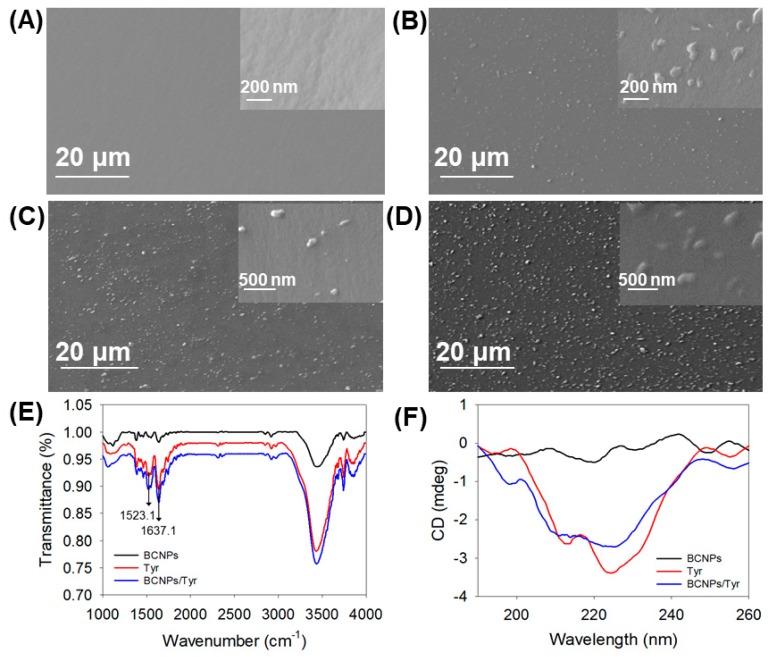
SEM images of (**A**) GCE, (**B**) BCNPs/GCE, (**C**) BCNPs/Tyr/GCE, (**D**) BCNPs/Tyr/Nafion/GCE. (**E**) FTIR spectra of BCNPs, Tyr and BCNPs/Tyr. (**F**) CD spectra of BCNPs, Tyr and BCNPs/Tyr.

The as prepared BCNP have high conductivity and are well dispersible in solution, so they could be utilized for the BCNP based enzyme biosensor (BCNPs/Tyr/Nafion/GCE) construction by simply incorporation of Tyr as signaling enzyme (Scheme 1). The surface morphology of the prepared BCNPs/Tyr/Nafion/GCE electrode was evaluated with SEM analysis. As indicated in Figure 1, in comparison to the pristine GCE with smooth appearance surface (Figure 1A), the BCNPs modified GCE electrode presented biochar nanoparticles with an average particle size of 245.4 nm spreading all over the electrode surface (Figure 1B). For the BCNPs/Tyr/GCE electrode as shown in Figure 1C, the biochar nanoparticles on the electrode surface deteriorated due to presence of Tyr. After treating with Nafion, the surface of BCNPs/Tyr/Nafion/GCE electrode became smoother and more homogeneous (Figure 1D), suggesting well formation of BCNPs and enzyme film on the GCE.

The functional group properties of the prepared materials were evaluated by the Fourier Transform Infrared spectra. As shown in Figure 1E, the peak at 1637.1 cm^−1^ for acylamide I is the absorption of –C=O stretching vibration of peptide linkages in the protein’s backbone. The peak at 1523.1 cm^−1^ for acylamide II is corresponding to the absorption of –N–H bending and C–N stretching. These two peaks are characteristic peaks of Tyr that are commonly used to determine whether Tyr retains its native conformation. Comparing to the native Tyr, the BCNPs/Tyr presented the same characteristic peaks of Tyr, indicating that Tyr still maintains its original conformation in the BCNPs/Tyr complex. The secondary structure of Tyr in the BCNPs/Tyr complex was further evaluated by circular dichroism spectra. As shown in Figure 1F, both Tyr and BCNPs/Tyr showed two negative bands which represent the presence of α-helix structures [42]. The bands of Tyr were located at about 212 and 224 nm while the characteristic bands of BCNPs/Tyr were presented approximately at 210 and 224 nm. There was only a small difference in the characteristic band positions of the two samples. In addition, it was calculated that the α-helix content in the Tyr sample was 27.5%, and the α-helix content in the BCNPs/Tyr was 25.0%. The little changes in these two samples indicated that there was almost no conformational change in the BCNPs/Tyr complex for Tyr, and it also demonstrated the good biocompatibility and environmental friendliness of BCNP.

### 3.2. Electrochemical Impedance Analysis

**Figure 2:**
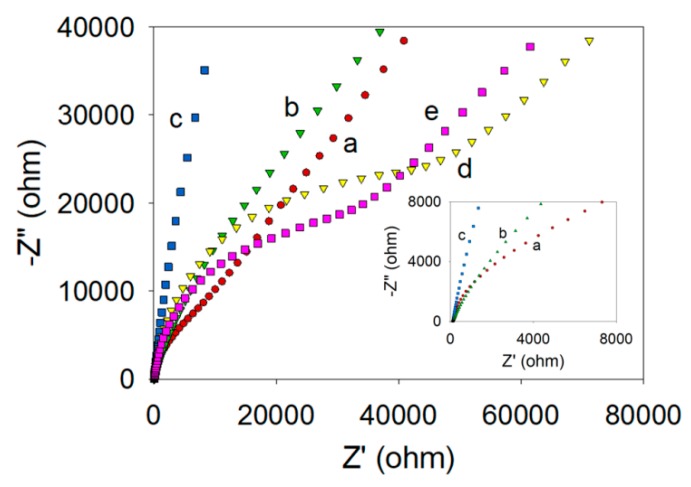
Nyquist plots of (**a**) GCE, (**b**) Nafion/GCE, (**c**) BCNPs/Nafion/GCE, (**d**) Tyr/Nafion/GCE, (**e**) BCNPs/Tyr/Nafion/GCE in 5 mM K_3_Fe (CN)_6_ containing 0.1 M KCl.

The electrochemical impedance measurements were utilized to detect the formation of each material on the GCE electrode and electron transfer properties of these electrodes. Figure 2 shows the electrochemical impedance spectroscopy (EIS) of a bare GCE, GCE modified with Nafion, BCNPs/Nafion/GCE, Tyr/Nafion/GCE and BCNPs/Tyr/Nafion/GCE in the 5 mM K_3_Fe(CN)_6_ solution containing 0.1 M KCl. As can be seen from the Figure 2, the impedance of bare GCE was 328.9 Ω. When the GCE was modified with Nafion, the Rct value was 0.0263 Ω, indicating that Nafion could both well protect the electrode material to keep it intact for a long time, and also was a good conductive film [43,44]. The Rct value at the BCNPs/Nafion/GCE electrode was 0.0169 Ω which was smaller than that of the Nafion/GCE electrode, indicating that the BCNP have greater conductivity. When tyrosinase was immobilized on the surface of GCE, the Rct of Tyr/Nafion/GCE increased obviously to 2242 Ω because of the increase of the thickness of the interface, which also proved that the introduction of tyrosinase effectively blocked the electron transfer between the electrode and [Fe(CN)_6_]3^−^/4^−^ [45]. In addition, BCNPs/Tyr/Nafion/GCE electrode had a relatively small resistance value of 1483 Ω in comparison, mainly due to the incorporation of the highly conductive BCNP.

### 3.3. Electrochemical Response of BCNP Enzyme Biosensor to BPA

**Figure 3:**
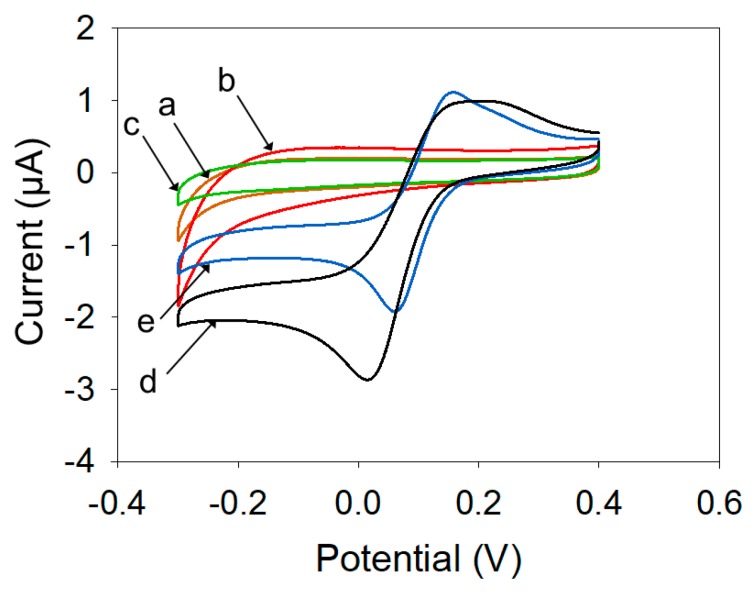
Cyclic voltammograms of the biosensor by using different electrodes (**a**–**c**) before and (**d**,**e**) after the addition of BPA (100 μM), (**a**) GCE, (**b**) BCNPs/Nafion/GCE, (**c**) BCNPs/Tyr/Nafion/GCE, (**d**) BCNPs/Tyr/Nafion/GCE, (**e**) Tyr/Nafion/GCE. Scan rate: 0.05 V s^−1^.

The electrochemical response of BCNP enzyme biosensor to bisphenol A was investigated by the cyclic voltammetry method, since the immobilized Tyr enzyme can react with BPA and produces electrochemically active quinones that can be electrochemically reduced to corresponding polyhydric phenol and produce a cathodic current [40]. As shown in Figure 3, without target BPA, there was no current peak observed with the GCE (Figure 3a), BCNPs/Nafion/GCE (Figure 3b) and BCNPs/Tyr/Nafion/GCE (Figure 3c). It was observed that the BCNPs modified GCE presented higher current signal compared to the GCE due to the presence of BCNPs, and after involving Tyr enzyme, the current signal was a little reduced, attributed to the presence of non-conductive enzyme molecules, suggesting well maintenance of Tyr. When BPA (100 µM) was added, a cathodic peak current at potential of 0.02 V was presented with the BCNPs/Tyr/Nafion/GCE, where the current change was 2.703 µA (Figure 3d), suggesting successful generation of electrochemical current response of the immobilized Tyr in the BCNP biosensor with the presence of target BPA. By using the Tyr/Nafion/GCE sensor (Figure 3e), the BPA reduction peak current can also be observed, but with less current change (1.771 µA) and at a higher potential of 0.06 V. This demonstrated that an enzyme biosensor constructed with BCNP could improve the electrochemical signal for BPA detection and also with lower reduction potential, which was attributed to the high conductivity and large specific surface area of the prepared BCNP. 

**Figure 4:**
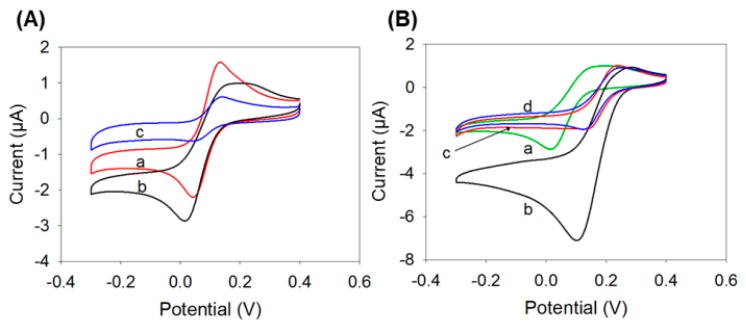
Cyclic voltammograms of the BCNPs/Tyr/Nafion/GCE biosensor in response to 100 μM BPA, (**A**) depending on various amounts of BCNP loading (a-c represent 0.25, 0.375 and 0.5 mg mL^−1^ of BCNP concentrations, respectively), and (**B**) depending on various amounts of Tyr loading (a–d represent 0.1, 0.5, 1.0 and 1.5 mg mL^−1^ of Tyr concentrations, respectively). Scan rate: 0.05 V s^−1^.

Amperometric current properties of the BCNPs/Tyr/Nafion/GCE biosensor in response to BPA with different loading amounts of BCNP and Tyr were assessed in detail. As indicated in Figure 4A, with the increase of BCNP from 0.25 to 0.5 mg mL^−1^ (Tyr was 0.1 mg mL^−1^), the reduction current response of the BCNPs/Tyr/Nafion/GCE biosensor to BPA increased firstly and then decreased. When BCNP concentration was 0.375 mg mL^−1^, the current signal was the largest. The current profiles of BCNP biosensor with various amounts of Tyr loaded, in response to BPA, are indicated in Figure 4B. As shown, the reduction currents increased with the elevation of the Tyr concentrations from 0.1 to 0.5 mg mL^−1^, and decreased with higher level of Tyr (BCNP was 0.375 mg mL^−1^), meanwhile, the reduction potentials were correspondingly increased from 0.02 (with 0.1 mg mL^−1^ of Tyr) to 0.08 V (with 0.5 mg mL^−1^ of Tyr). The reason for these phenomena was that greater concentrations of BCNP or Tyr resulted in the thicker films of the electrode surface, which led to an increase of interfacial electron transfer resistance [46]. Therefore, BCNPs/Tyr/Nafion/GCE biosensor prepared with 0.375 mg mL^−1^ of BCNP and 0.5 mg mL^−1^ of Tyr were used in the following study. 

For comparison, other enzyme biosensors including GN/Tyr/Nafion/GCE, MWNTs/Tyr/Nafion/GCE and GP/Tyr/Nafion/GCE that were prepared using graphite nanoparticle (GN), multi-wall carbon nanotube (MWNT) and graphene (GP), respectively, and their electrochemical properties were assessed. As shown in Figure S1A, electrochemical impedance spectroscopy indicated that the conductivity of the enzyme biosensor with BCNP was higher than enzyme biosensors prepared using GN and GP, and lower than with the MWNTs, i.e., MWNTs/Tyr/Nafion/GCE > BCNPs/Tyr/Nafion/GCE > GP/Tyr/Nafion/GCE > GN/Tyr/Nafion/GCE. From the amperometric current profiles in response to the same amounts of BPA in Figure S1B, it can be found that the enzyme biosensor with BCNP presented the largest current signal, where the order of the current change values was: BCNPs/Tyr/Nafion/GCE > GP/Tyr/Nafion/GCE > GN/Tyr/Nafion/GCE > MWNTs/Tyr/Nafion/GCE. One reason why the enzyme biosensor with BCNP presented the largest response signal to the same concentration of BPA is due to the good conductivity property of the proposed BCNP, and another possible factor is that the biocompatible characteristics of the plant derived biochar can make the biocatalytic property of the enzyme well maintained. In addition, the BCNP presented a larger surface area of 321.68 m^2^ g^−1^ than the graphite nanoparticle (270.15 m^2^ g^−1^), the graphene (11.91 m^2^ g^−1^) and the MWNTs (247.80 m^2^ g^−1^), that may have also contributed to improved current responses. 

### 3.4. Sensitivity of BCNP Enzyme Biosensor

**Figure 5:**
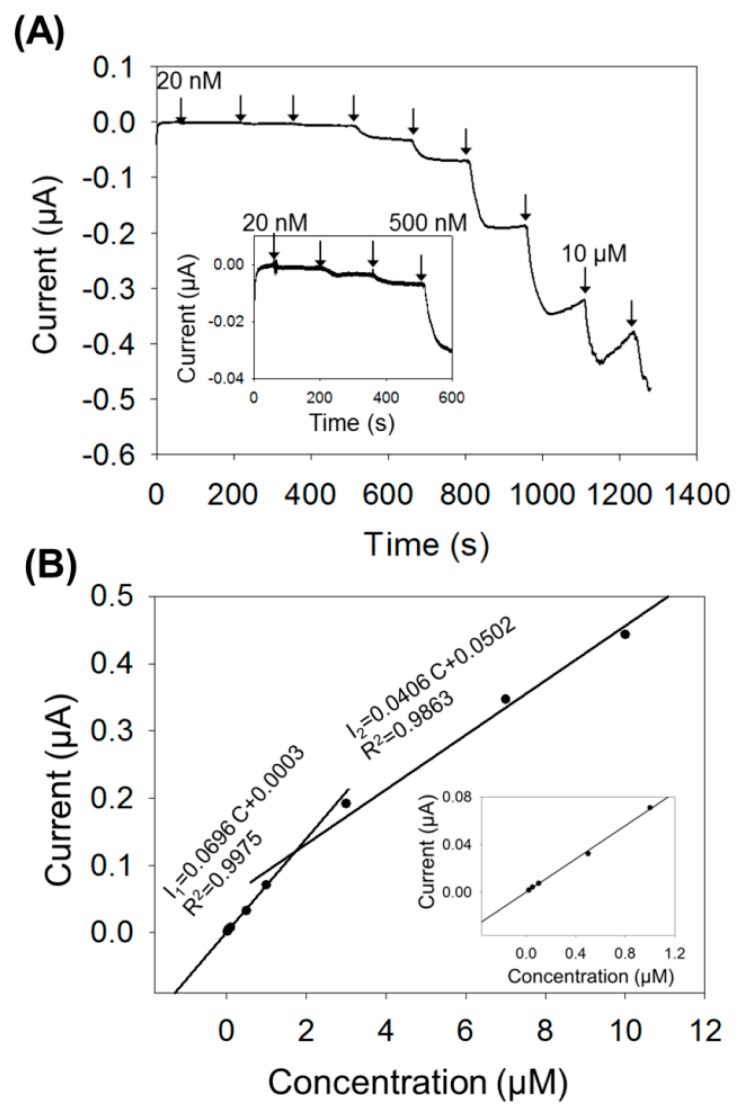
(**A**) Time–current responses of BCNPs/Tyr/Nafion/GCE biosensor for detecting various concentrations of BPA (BCNP 0.375 mg mL^−1^, Tyr 0.5 mg mL^−1^) in 10 mL PB solution at 0.08 V. (Inset: Time–current responses of the BCNPs/Tyr/Nafion/GCE biosensor for detecting BPA at low concentrations.) (**B**) Calibration curve for BPA sensing by plotting the current values versus BPA concentration. The BPA concentrations are 20, 50, 100 and 500 nM and 1, 3, 7, 10 and 15 μM, respectively.

In order to accurately and quantitatively detect BPA, the amperometry detection of BPA was performed with the optimized BCNPs/Tyr/Nafion/GCE biosensor (prepared with 0.375 mg mL^−1^ of BCNP and 0.5 mg mL^−1^ of Tyr). A typical amperometric I–t curve of BCNPs/Tyr/Nafion/GCE biosensor upon addition of specific amounts BPA solution to PB (50 mM, pH 7.0) with potential of 0.08 V (Figure S2) and under constant stirring was obtained. As can be seen from the Figure 5A, the reduction current of the BCNPs/Tyr/Nafion/GCE biosensor increased as the BPA concentration increased from 0.02 to 10 μM. The current values were linearly correlated with the BPA concentrations, with the linear regression equation of $I_{1}=0.0696C+0.0003$ (*R*^2^ = 0.9975) from 0.02 to 1 μM of BPA and $I_{2}=0.0406C+0.0502$ (*R*^2^ = 0.9863) from 1 to 10 μM of BPA for BCNPs/Tyr/Nafion/GCE (Figure 5B). In addition, the sensitivity of the BCNPs/Tyr/Nafion/GCE electrode was 0.985 µA µM^−1^ cm^−2^ when the BPA concentrations were 0.02–1 μM, and as BPA concentrations were 1–10 μM, the sensitivity of this electrode was 0.575 μA μM^−1^ cm^−2^. The corresponding detection limit of the prepared BCNPs/Tyr/Nafion/GCE biosensor was 3.18 nM. Thus, these results fully proved that BCNP could improve the electrochemical biosensor detection performance, and this was mainly due to the excellent conductivity and good biocompatibility of BCNP. The analytical characteristics of the BCNPs/Tyr/Nafion/GCE biosensor are summarized in Table S1, in comparison with other sensors for bisphenol A detection reported in the literature. By comparison, it was further proved that the biosensor proposed in this study had great advantages.

### 3.5. Kinetic Constant, Reproducibility, Stability and Selectivity

**Figure 6:**
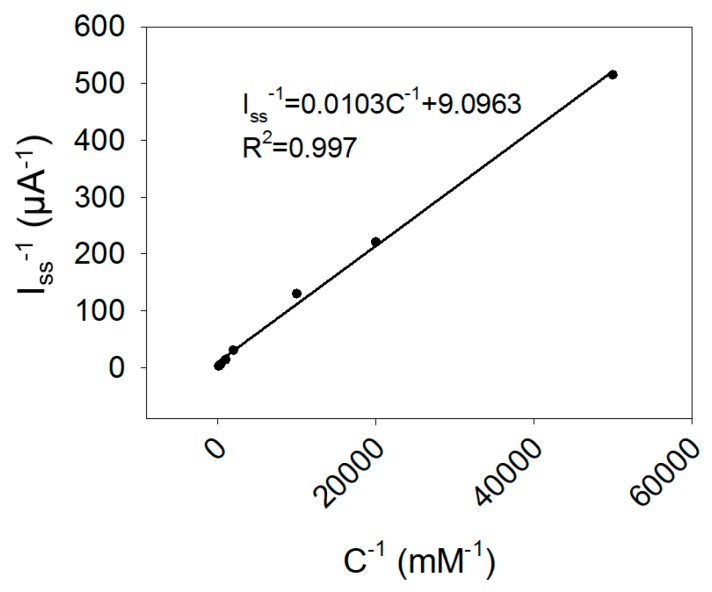
Lineweaver–Burk plots of the BCNPs/Tyr/Nafion/GCE electrode.

The Michaelis–Menten constant (*K*_m_) is the substrate concentration at which the enzymatic reaction rate is half of the maximum reaction rate, and it is also one of the most important kinetic parameters. In electrochemistry, the equation for measuring the Michaelis constant is $I_{\mathrm{ss}}^{-1}=1/I_{\max}{+K}_{m}/(I_{\max}C)$, where *I*_ss_ is the response current after addition of a certain concentration of substrate, *I*_max_ is the maximum current when the substrate is saturated and *C* is the substrate concentration [47]. Kinetic parameter *K*_m_ was evaluated by the Lineweaver–Burk plot using BPA as substrate (Figure 6). When calculated, the *K*_m_ value was 1.13 μM, which was much smaller than the values reported in other literatures [48,49,50], indicating that the biosensor could maintain its good enzymatic activity, and also had a high affinity for BPA.

The reproducibility of the BCNPs/Tyr/Nafion/GCE biosensor was studied by determining the response to 3 μM BPA by six different electrodes prepared independently. The RSD was found to be 7.28%, demonstrating that the biosensor constructed in this paper had excellent reproducibility. Multiple cycle detection ability was also investigated, and found the proposed biosensor could perform four times repeated measurement of same amount of BPA with negligible signal lose (RSD of 5.74%). Additionally, the storage stability of the BCNPs/Tyr complex solution was also further accessed by measuring amperometric response to 3 μM BPA for every 7 days. During the measurement, the BCNPs/Tyr complex solution was stored at 4 °C. This result showed that the current response of the electrode maintained 86.9% of its initial response after one month which indicated that the composite solution had good stability. This also means that in the actual testing, only the preparation of the electrode is required and there is no need to configure a fresh solution before preparing the electrode, which saves a lot of time and cost.

**Figure 7:**
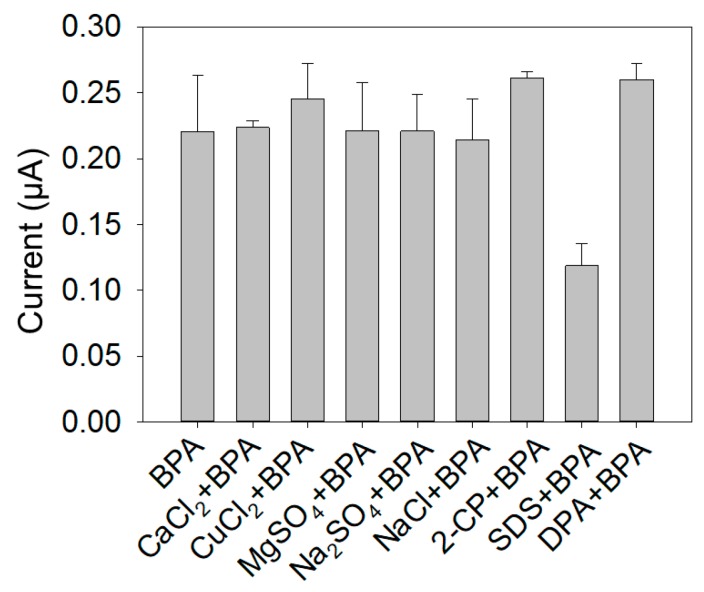
Specificity of the BCNPs/Tyr/Nafion/GCE biosensor for detection of BPA (3 μM) in 50 mM PB (PH = 7.0), where other irrelevant compounds coexist individually including 3 mM of CaCl_2_, CuCl_2_, MgSO_4_, Na_2_SO_4_, NaCl, 2-CP, SDS and DPA.

Selectivity of the biosensor toward 3 μM BPA in the presence of various ionic interferents (with 100-fold concentration higher than BPA) that may be present in water was assessed. As shown in Figure 7, with co-presence of the ions including Cl^−^, SO_4_^2−^, Ca^2+^, Mg^2+^, the BPA detection signals were not affected, suggested there is no interference. But, when there was Cu^2+^, the current signal response to BPA was increased for 11.10 % compared to the control, this is because Cu^2+^ can promote the enzyme reaction as reported previously [51]. The BPA detection signals in the presence of other phenolic compounds were also evaluated. As indicated, both 2-chlorophenol and diphenolic acid would slightly increase the current signal (18.31% and 17.76% increase), that is because tyrosinase enzyme can also catalytically convert of the two phenolic compounds to electroactive products. It was also found that the anionic surfactant, sodium dodecyl sulfate, could reduce the current signal of BPA detection (46.26 % reduction) due to inhibition of enzyme activity by the sodium dodecyl sulfate [52].

### 3.6. Real Sample Detection

To verify the feasibility of the BCNPs/Tyr/Nafion/GCE biosensor constructed for real environmental water detection, the fresh groundwater samples were added to a concentration of BPA and then diluted 100-fold with PB for detection. Under optimal conditions, we quantified the concentrations of BPA in the treated water samples by amperometric I–t curves. As can be seen in Table 1, the BCNPs/Tyr/Nafion/GCE biosensor electrochemical measurements of the treated water samples showed 96.67%, 108.60% and 106.14% recoveries with relative standard deviations of 2.14%, 1.05% and 2.25%. In addition, the accuracy of this biosensor was further verified by performing HPLC methods to detect the same concentration of BPA in groundwater. The recoveries of treated water samples measured by HPLC method were 96.67%, 119.20% and 99.57% for 3, 5 and 7 μM BPA, which indicated that the BCNPs/Tyr/Nafion/GCE biosensor electrochemical measurements were in good agreement with high-precision HPLC measurements. The results of the above analysis indicate that the BCNPs/Tyr/Nafion/GCE biosensor prepared in this article has great potential for the detection of actual water samples due to its high sensitivity, fast response time, low resistance, low cost, simple operation and ability to measure on site. 

## 4. Conclusions

In this article, we used a Nafion embedding method to prepare a novel biochar nanoparticle-based tyrosinase biosensor for the detection of bisphenol A. This was the first time that biochar had been proposed for use in conjunction with enzymes in the biosensor. The prepared biochar nanoparticles had large specific surface areas, good conductivity, biocompatibility and environmental friendliness which were beneficial to enhance the activity of the enzyme. More importantly, the prepared BCNPs/Tyr/Nafion/GCE biosensor had high sensitivity, reproducibility, selectivity, a low *K*_m_ value, and also proved its successful application in real environmental water detection. Therefore, owning to the easy preparation of electrodes, simple system operation, and superior detection performances, the BCNP based enzyme biosensor would have great prospective for application in sensing. 

## Figures and Tables

**Table 1:** Determination of BPA in real ground water samples using BCNPs/Tyr/Nafion/GCE biosensor and HPLC method.

Added (μM)	Determined by BCNPs/Tyr/Nafion Electrochemical Biosensor			Determined by HPLC	
	Detected (μM) ^a^	RSD (%)	Recovery (%)	Detected (μM) ^a^	Recovery (%)
**3**	2.90	2.14	96.67	2.90	96.67
**5**	5.43	1.05	108.60	5.96	119.20
**7**	7.43	2.25	106.14	6.97	99.57

^a^ Mean of three measurements.

## Supplementary Materials

Supplementary Materials can be found at https://www.mdpi.com/1424-8220/19/7/1619/s1.
