# Supplementary material for: Electrochemical Enzyme Biosensor Bearing Biochar Nanoparticle as Signal Enhancer for Bisphenol A Detection in Water
Source: Sensors (Basel). 2019 Apr 4;19(7):1619. doi: 10.3390/s19071619 (PMC6479578; doi:10.3390/s19071619)
Supplement: Supplementary file 1 [file sensors-19-01619-s001.pdf]

Electrochemical enzyme biosensor bearing biochar nanoparticle as signal enhancer for bisphenol A detection in water

Yang Liu, Lan Yao, Lingzhi He, Na Liu and Yunxian Piao*

Key Laboratory of Groundwater Resources and Environment (Jilin University), Ministry of Education, Jilin Provincial Key Laboratory of Water Resources and Environment, College of New Energy and Environment, Jilin University, Changchun 130021, China

*__Corresponding author:__ Professor Yunxian Piao

Key Laboratory of Ground Water Resources and Environment of the Ministry of Education, College of New Energy and Environment, Jilin University, 2519 Jiefang Road, Changchun 130021, China

E-mail: yxpiao@jlu.edu.cn, Phone: +86-18604414674, Fax: +86-431-8850-2606

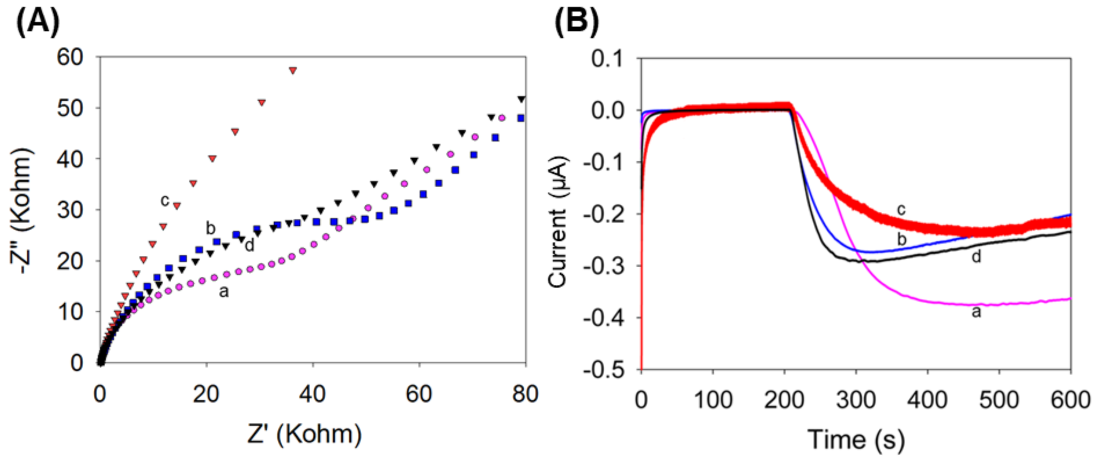

Fig. S1 (A) Nyquist plots of the four different biosensors in 5 mM $K_3Fe\ (CN)_6$ containing 0.1 M KCl. (B) The amperometric current profiles of the four biosensors in response to the same amounts of BPA (3 μM) with the potential of 0.08 V versus Ag/AgCl. The four biosensors are: (a) BCNPs/Tyr/Nafion/GCE, (b) GN/Tyr/Nafion/GCE, (c) MWNTs/Tyr/Nafion/GCE, (d) GP/Tyr/Nafion/GCE.

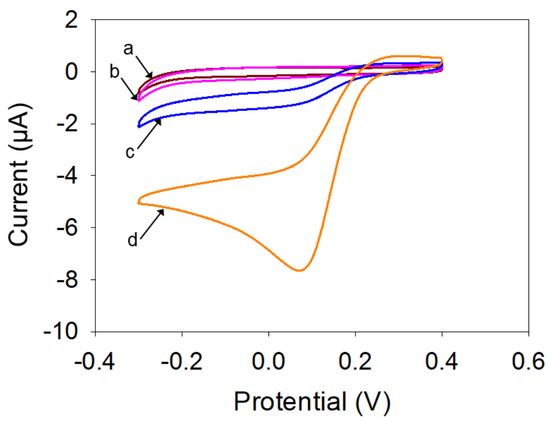

Fig. S2 Cyclic voltammograms of BCNPs/Tyr/Nafion/GCE (BCNPs 0.375 mg mL^{-1}, Tyr 0.5 mg mL^{-1}) electrode in blank (a) and different concentrations of BPA: (b) 1 µM, (c) 10 µM, (d) 100 µM in PB (50 mM, PH 7.0) with scan rate 50 mV s^{-1}.

Table S1 Comparison of analytical characteristics toward bisphenol A for reported biosensors

**Biosensor**	**Sensitivity [μA mM^{-1} cm^{-2}]**	**Linear range [μM]**	**LOD [μM]**	**Reference**
Tyr–AuNPs/SPCE	419	0.042–36	0.01	[1]
Tyr–NiNPs/SPCE	758	0.91–48	0.0071	[1]
Tyr–Fe_3O_4/SPCE	544	0.027–40	0.0083	[1]
Tyr–polylysine-SWCN/GCE	788	0.004–11.48	0.00097	[2]
Laccase-CB/SPE	70.8	0.5–50	0.2	[3]
Tyr–SWCP–CPE	138	0.1-12	0.02	[4]
GN-CNT/GCE	-	0.06–10	42	[5]
MNPs-rGO/GCE	18.1	0.06-11	0.17	[6]
BCNPs/Tyr/Nafion/GCE	985	0.02-10	0.00318	This work

References

[1] Alkasir, R.S.; Ganesana, M.; Won, Y.H.; Stanciu, L.; Andreescu, S.; Enzyme functionalized nanoparticles for electrochemical biosensors: a comparative study with applications for the detection of bisphenol A, Biosens. Bioelectron 2010, 26 (1), 43-49. [CrossRef]

[2] Han, M.; Qu, Y.; Chen, S.; Wang, Y.; Zhang, Z.; Ma, M.; Wang, Z.; Zhan, G.; Li, C.; Amperometric biosensor for bisphenol A based on a glassy carbon electrode modified with a nanocomposite made from polylysine, single walled carbon nanotubes and tyrosinase, Microchim. Acta 2013, 180 (11-12), 989-996. [CrossRef]

[3] Portaccio, M.; Di Tuoro, D.; Arduini, F.; Moscone, D.; Cammarota, M.; Mita, D.G.; Lepore, M.; Laccase biosensor based on screen-printed electrode modified with thionine–carbon black nanocomposite, for Bisphenol A detection, Electrochimica Acta 2013, 109, 340-347. [CrossRef]

[4] Mita, D.G.; Attanasio, A.; Arduini, F.; Diano, N.; Grano, V.; Bencivenga, U.; Rossi, S.; Amine, A.; Moscone, D.; Enzymatic determination of BPA by means of tyrosinase immobilized on different carbon carriers, Biosens. Bioelectron 2007, 23 (1), 60-65. [CrossRef]

[5] Zheng, Z.; Du, Y.; Wang, Z.; Feng, Q.; Wang, C.; Pt/graphene-CNTs nanocomposite based electrochemical sensors for the determination of endocrine disruptor bisphenol A in thermal printing papers, Analyst 2013, 138 (2), 693-701. [CrossRef]

[6] Zhang, Y.; Cheng, Y.; Zhou, Y.; Li, B.; Gu, W.; Shi, X.; Xian, Y.; Electrochemical

sensor for bisphenol A based on magnetic nanoparticles decorated reduced graphene oxide, Talanta 2013, 107, 211-218. [CrossRef]
